# Geschichtsbewusstsein und historische Verantwortung in der deutschen Humangenetik

**DOI:** 10.1515/medgen-2021-2079

**Published:** 2021-08-14

**Authors:** M. Krischel, H. Fangerau, F. Söhner

**Affiliations:** Institut für Geschichte, Theorie und Ethik der Medizin, Centre for Health and Society, Medizinische Fakultät, Heinrich-Heine-Universität Düsseldorf, Moorenstraße 5, 40225 Düsseldorf, Deutschland

**Keywords:** Medizinische Genetik, Medizingeschichte, Oral History, Zeitgeschichte, Deutsche Gesellschaft für Humangenetik, medical genetics, history of medicine, oral history, contemporary history, German society for human genetics

## Abstract

Historiker*innen haben immer wieder auf personelle und institutionelle Kontinuitäten zwischen der Eugenik in Deutschland vor 1945 und der sich entwickelnden Humangenetik im Nachkriegsdeutschland hingewiesen. Wie aber wurde diese äußere Wahrnehmung unter deutschen Humangenetiker*innen diskutiert und spielte sie für das Selbstbild der Disziplin eine Rolle? Auf der Basis von gedruckten Quellen und biographischen Interviews wurden das Geschichtsbewusstsein und die Übernahme historischer Verantwortung unter deutschen Humangenetiker*innen untersucht, die in der Zeit zwischen den 1970er und den 2010er Jahren im Feld tätig waren. Ein historisches Bewusstsein spielte in der Erinnerung der Zeitzeug*innen schon vor den 1980er Jahren eine Rolle für ihr Fach und seit der Gründung der Deutschen Gesellschaft für Humangenetik im Jahr 1987 wird in der Gesellschaft die Frage der Form einer historischen Verantwortungsübernahme immer wieder diskutiert. Sie findet ihren praktischen Ausdruck z. B. in Diskussionen mit der Öffentlichkeit und in der Umbenennung von Preisen.

## Abstract (en)

Historians have repeatedly pointed to personal and institutional continuities between eugenics in Germany before 1945 and the developing human genetics in post-war Germany. But how was this external perception discussed among German human geneticists and did it play a role in the discipline’s self-image? On the basis of printed sources and biographical interviews, the historical consciousness and the assumption of historical responsibility among German human geneticists active in the field between the 1970s and the 2010s were investigated. In the memories of the contemporary witnesses, historical awareness played a role for their discipline even before the 1980s, and since the founding of the Deutsche Gesellschaft für Humangenetik in 1987, the question of the form of showing historical responsibility has been discussed repeatedly. It has been expressed, for example, in discussions with the public and renaming of prizes.

## Einleitung

Im Jahr 1988, ein Jahr nach der Gründung der Deutschen Gesellschaft für Humangenetik (GfH), publizierte Peter Emil Becker den ersten Band seines Werkes zur Geschichte der Eugenik in Deutschland. [1] Mit diesem Buch wollte er anhand von Persönlichkeitsprofilen – darunter sein Göttinger Vorgänger Fritz Lenz – die „Wege ins Dritte Reich“ und die Beteiligung von Anthropologen und biomedizinischen Wissenschaftlern am eugenischen Denken und Handeln diskutieren. Die Auseinandersetzung eines prominenten Humangenetikers mit der nicht immer unproblematischen Geschichte seines Fachs erfuhr viel Aufmerksamkeit und Anerkennung. Gleichzeitig war Beckers Buch nicht unumstritten, weil die Frage im Raum stand, ob eine Perspektive aus der Humangenetik heraus zu einer unvoreingenommenen Geschichtsschreibung führen könne. [2] Später wurde Becker vorgeworfen, seine Mitgliedschaft in der NSDAP verschwiegen zu haben. [3] In der Folge wurde ein ab 1998 von der Gesellschaft für Neuropädiatrie vergebene „Peter-Emil-Becker-Preis für herausragende Leistungen auf dem Gebiet der Neuropädiatrie“ umbenannt. [4]

Mehr als 30 Jahre nach der Veröffentlichung von Beckers Buch stellen sich immer noch Fragen nach dem Selbstverständnis der Humangenetik in Deutschland und ihrer Rolle in der Medizin im Nationalsozialismus (NS). Mit diesen Fragen ist die Humangenetik nicht allein. In den letzten Jahren erschienen verschiedene Übersichtsarbeiten, die sich der NS-Vergangenheit unterschiedlicher medizinischer Fachgesellschaften angenommen haben. [5] Hierbei werden Fragen nach Geschichtsbewusstsein, historischer Verantwortung und Erinnerungskultur berührt. [6]

Zahlreiche schon vor 1945 im Feld der Vererbungslehre und Rassenhygiene beschäftigte Personen konnten in der BRD nach meist relativ kurzen Unterbrechungen als Humangenetiker*innen weiterwirken [7], [8]. Benoît Massin deutete die fachinterne Auseinandersetzung der Genetiker*innen mit ihrem Fach entsprechend als eine „*apologetische Geschichtsschreibung*“. [9] Erst seit den 1990er Jahren überwiegt der fachfremde historische Blick auf die Humangenetik gegenüber einer Geschichtsschreibung aus dem Fach heraus. [8] Die Auseinandersetzung der Nachkriegsgeneration der Humangenetik mit ihrer Geschichte kommt in dieser Historiographie nur am Rande vor. Unsere Studie fokussiert auf den Umgang mit der Geschichte genau dieser Generation. Der Beitrag untersucht, wie die deutschen Humangenetiker*innen seit den 1970er Jahren, d. h. im Zuge oder nach dem ersten großen Generationenwechsel der Nachkriegszeit an den deutschen Universitäten, mit der Geschichte ihres Fachs umgegangen sind bzw. ob und wenn ja welche Wege sie gesucht haben, diese Geschichte zu adressieren. Zu diesem Zweck wurden aus dem Fach heraus veröffentlichte Schriftquellen zur Geschichte der Humangenetik in Deutschland (kulturelles Gedächtnis) mit Erinnerungen von Zeitzeug*innen (soziales Gedächtnis) vergleichend ausgewertet. [10] Besonderes Augenmerk wurde dabei auf Konjunkturen von Schweigen und Erinnern in der Bundesrepublik, auf erinnerungskulturelle Rituale und die durch Akteur*innen und Institutionen ausgedrückte Wahrnehmung einer historischen Verantwortung gelegt. [11], [12]

## Was sind Geschichtsbewusstsein und Historische Verantwortung?

Jenny Tillmanns hat die These aufgestellt, dass historische Verantwortung sich nicht in erster Linie auf die Taten der Vorfahren bezieht, sondern auf hier und heute gelebte „*historische Identität* (*wer wir sind*) *und* […] *historische Praxis* (*was wir tun*)“. [13] Da sich historische Identität auf die historische Herkunft beziehe, lasse sich historische Verantwortung durch die Geschichtlichkeit eines Individuums oder einer Gemeinschaft begründen.

In Anlehnung an Larry May [14] bezieht sich historische Verantwortung auf die von den Vorgängern begangenen Taten oder Untaten. Entscheidend sei in diesem Zusammenhang die Anerkennung, dass Einzelne zwar keinen Einfluss auf ihre historische Zugehörigkeit haben, jedoch auf den Umgang mit der Geschichte, wenn sie mit ihr konfrontiert werden. Im so verstandenen Sinne bezieht sich ‚historische Verantwortung‘ im Wesentlichen auf ‚unseren Umgang mit unserer Geschichte‘.

Im Falle der deutschen Humangenetik bezieht sich dieser Umgang konkret auf die Auseinandersetzung mit der Rassenanthropologie und dem ‚wissenschaftlichem Rassismus‘ sowie mit der Rassenhygiene – als deutschem Begriff, der weitgehend deckungsgleich mit dem internationalen Begriff der Eugenik ist – sowie der Zwangssterilisationen im NS.

## Material und Methoden

Um das Geschichtsbewusstsein und das Ausmaß einer daraus abgeleiteten fachbezogenen historischen Verantwortung unter deutschen Humangenetiker*innen zu ermitteln, wurden Veröffentlichungen von Fachvertreter*innen und Geschichtsforschenden sowie Stellungnahmen der GfH seit ihrer Gründung 1987 analysiert. Um diese Perspektive durch persönliches Erleben und Erinnern der Nachkriegsgeneration zu ergänzen, wurden 29 biographische Interviews mit deutschsprachigen Humangenetiker*innen ausgewertet. [15]

Die schriftlichen Quellen wurden heuristisch-qualitativ mit Hinblick auf die Forschungsfragen, die Interviews mit den Methoden der qualitativen Inhaltsanalyse und Grounded Theory ausgewertet. [15]

## Kulturelles Gedächtnis in der deutschen Humangenetik

Die Beschäftigung mit der eigenen (eugenischen) Geschichte begann in der Humangenetik in den ausgehenden 1960er Jahren. Weingart et al. schildern die Reflexion der eigenen Geschichte des Faches und die folgende ethische Verantwortung der Disziplin auf dem ‚Forum Philippinum‘ in Marburg 1969 [16] und werten sie als mehr *„als nur die längst überfällige Vergangenheitsbewältigung. Es war zugleich die Etablierung eines dauerhafteren innerfachlichen Diskurses über die ethischen Verpflichtungen und Grenzen der zukünftigen Humangenetik*.“ [7]

Neben der eingangs diskutierten Veröffentlichung Peter Emil Beckers gehört zu den außerhalb des Fachs wahrgenommenen [17] Initialzündungen der Auseinandersetzung der Humangenetiker*innen mit ihrer eugenischen Vergangenheit eine Publikation von Benno Müller-Hill. Er setzte sich in seiner Studie „Tödliche Wissenschaft“ kritisch mit der Rolle der Anthropologie und Humangenetik in der NS-Rassenpolitik auseinander. [18] Müller-Hill, z. Z. der Veröffentlichung Professor für Genetik an der Universität Köln, wies in seiner Studie auf die wesentliche Rolle des Kaiser-Wilhelm-Instituts für Anthropologie in der Erbgesundheits- und Rassenpolitik des „Dritten Reiches“, insbesondere auch in der Judenpolitik, hin. Er stellte heraus, dass der *„Aufstieg der Genetik durch einen gigantischen Prozeß der Unterdrückung ihrer Geschichte charakterisiert“* [18] sei. Mit dieser Einsicht war Müller-Hill 1987 seiner Zeit voraus. Typischer für die Attitüde der seinerzeitigen deutschen Ärzteschaft und Wissenschaft erscheint – allen laufenden Debatten zum Trotz – eine Einschätzung des Nobelpreisträgers, ehemaligen NSDAP-Mitglieds und ehemaligen Präsidenten der Max-Planck-Gesellschaft Adolf Butenandt. Während der Vorbereitungsphase zum Buch äußerte Butenandt in einem Schreiben an den Sohn Otmar von Verschuers er habe „*kein gutes Gefühl über die Absichten des Herrn Müller-Hill und fürchte, er wird einige unberechtigte und längst ausgeräumte Auffassungen der Nachkriegszeit neu beleben und gegen die Kaiser-Wilhelm*/*Max-Planck-Gesellschaft den Vorwurf mangelnder Verantwortung erheben*.“ [19] Müller-Hill bemerkte, dass die Geschichte der NS-Rassenpolitik unter Anthropologen und Humangenetikern in den ersten 25 Jahren nach dem Ende des Zweiten Weltkriegs „*als störend und uneigentlich behandelt*“ [18] angesehen wurde.

### Stellungnahmen der GfH

Seit ihrer Gründung hat die GfH auch die Geschichte der Humangenetik zu ihrer Angelegenheit gemacht. Bereits anlässlich ihres ersten Kongress 1989 bildete die Gesellschaft eine ‚Kommission für Öffentlichkeitsarbeit und ethische Fragen‘. Diese gab eine Erklärung ab, die in der ersten Ausgabe der neuen Zeitschrift der Gesellschaft, der medizinischen genetik, abgedruckt wurde. Im Text wird aus einem „*Bewußtsein der besonderen Verantwortung vor dem Hintergrund der Geschichte unseres Faches* […] *eine Anwendung humangenetischer Untersuchungsmethoden mit eugenischer Zielsetzung*“ entschieden abgelehnt. Maßstab der Tätigkeit der Gesellschaft bleibe „*das Wohl des Einzelnen und seiner Familie*“. Zudem kündigt die zum ersten Mal tagende Gesellschaft an, *„sich an der öffentlichen Diskussion über die humangenetische Forschung und Praxis zu beteiligen*“. [20]

Noch deutlicher wird ein Positionspapier der GfH aus dem Jahr 2007. Es enthält einen Abschnitt zu „*Prinzipien der modernen Humangenetik vor ihrem geschichtlichen Hintergrund“*. Die Geschichte des Faches wird hier in die Professionsethik integriert, wenn aus der „*spezifischen Geschichte der Humangenetik in Deutschland*“ in der „*Zeit des Nationalsozialismus*“ eine besondere Verantwortung abgeleitet wird. So heißt es: „*Humangenetiker sind sich ihrer besonderen Verantwortung bewusst, der Wiederholung solcher Entwicklungen entgegenzuwirken*.“ [21]

Als im Jahr 2008 der International Congress of Genetics in Berlin ausgerichtet wurde, fiel dies mit dem 75. Jahrestag der Verkündung des ‚Gesetzes zur Verhütung erbkranken Nachwuchses‘ (GVeN) zusammen. Zu diesem Anlass erschien in der medizinischen genetik eine Erklärung der GfH und der Deutschen Gesellschaft für Genetik. Das GVeN wird als „*Grundlage für eine systematische und gewalttätige Missachtung fundamentaler Menschenrechte*“ beschrieben, seine „*wahren Motive lagen in der biologistischen Auslese-Ideologie*“. Es wird festgehalten, dass an der *„inhaltlichen Vorbereitung und pseudowissenschaftlichen Begründung des Gesetztes, sowie an der Ausführung der Zwangsmaßnahmen* […] *deutsche Ärzte und Wissenschaftler maßgeblich beteiligt*“ waren. Auch „*Humangenetiker*“ hätten „*schwere Schuld auf sich geladen*“. Die Zeit nach 1945 sei „*zunächst von personellen und ideologischen Kontinuitäten geprägt*“ gewesen, erst „*in den sechziger und siebziger Jahren und insbesondere mit der Gründung der Deutschen Gesellschaft für Humangenetik* (*GfH*) *1989 vollzog sich ein bewusster Neuanfang des Faches*.“ In dieser Passage grenzt sich die Autorenschaft klar von den Eugenikern ab, die das Fach (mit-)prägten. Sie stilisieren die Gründung ihrer Fachgesellschaft zum Bruch mit der Kontinuität zur Zeit vor 1945. Die Erklärung schließt mit einem ausdrücklichen Bekenntnis zu historischer Verantwortung. *„Im Bewusstsein ihrer historischen Verantwortung bekennen sich die Mitglieder der Deutschen Gesellschaft für Humangenetik zu ihrer Verpflichtung, in ihrem ärztlichen und wissenschaftlichen Wirken wie auch im öffentlichen Diskurs für den Respekt vor allen Menschen in ihrer natürlichen genetischen Verschiedenheit einzutreten. Dies bedeutet insbesondere eine Absage an jede Form von Diskriminierung aufgrund ethnischer Merkmale oder aufgrund von genetisch bedingter Krankheit oder Behinderung*.“ [22]

Dass ein solches Bewusstsein in der deutschen Humangenetik zu diesem Zeitpunkt auch über den Jahrestag hinaus etabliert war, zeigt u. a. die Stellungnahme zur „*Eugenische[n] Argumentation im Beschluss des Bundesverfassungsgerichts zum Inzestverbot*“, die in der unmittelbar vorausgehenden Ausgabe der medizinischen genetik erschienen war. Die Autor*innen halten die „*eugenische Begründung* […] *für nicht akzeptabel*“ und empfehlen „*nachdrücklich, auf der Ebene höchstrichterlicher Rechtsprechung auf eugenische Begriffe und Argumentationen zu verzichten. Diese sind sachlich falsch und leisten darüber hinaus der Diskriminierung von Menschen und Familien Vorschub, die ohnehin ein schweres Schicksal haben*.“ [23]

Zuletzt bezeichnete der damalige Vorsitzende Klaus Zerres in seinem Vorwort zu einem 2014 zum 25. Kongress der GfH herausgegebenen Band „*[e]thische Fragen als permanente Herausforderung*“. Er verwies auf die Stellungnahme von 1989, in der eugenische Gesundheitspolitik ausdrücklich abgelehnt worden war und betont, dies gelte nicht nur weiter, sondern müsse auch weiter kommuniziert werden. [24]

## Zeitzeug*innen

Eine Reihe der befragten Zeitzeug*innen äußerten sich zu Fragen des Erinnerns an die Medizin im Nationalsozialismus, Eugenik und daraus abgeleitete historische Verantwortung in der deutschen Humangenetik.

Eine Person etwa berichtete über ein aktives Verschweigen in Fach und Gesellschaft in der Nachkriegszeit, das auch in die eigene Erinnerung fortgewirkt habe:

„*Das ist dann unter den Tisch gekehrt worden. Aber das war das einzige. Ich persönlich habe dazu gar keine Beziehung mehr. Die Zeit war ja völlig vorbei, als ich, ich meine, ich habe ja nach dem Krieg erst studiert. Nach der Nazi-Zeit. Ich […] weiß das nur aus den Berichten.*“

[25]

Mehrere Aussagen deuten auf einen Zusammenhang zwischen historischem Bewusstsein und ihrer Institutswahl hin:

„*[Ein Mentor] hat ganz klar gesagt: ‚Jeder in Deutschland der 1945 älter als 18 war, ist für mich verdächtig bis zum Beweis des Gegenteils. Das heißt, du kannst jetzt nur gehen zu [Widukind] Lenz in Hamburg, zu Vogel in Heidelberg und zu Weicker in Bonn. Das sind die drei […] von denen ich weiß, dass Sie sauber sind. Nur da kannst du hingehen.‘*“

[26]

Die Konfrontation mit der Vergangenheit durch Benno Müller-Hill wurde positiv wie negativ erinnert. Offen muss dabei bleiben, inwieweit die historische Einordnung seines Buches und die Erinnerung der Zeitzeugen an ihn ein sich selbst verstärkendes und verfestigendes System bildeten. Mehrere Befragte erwähnten, dass es auch sein unkonventionelles Auftreten gewesen sei, das unter einigen Fachvertreter*innen zu Ablehnung geführt habe. In einem Interview wird ausgeführt:

„*In seiner Festrede hatte Benno Müller-Hill […] ein für damalige Verhältnisse sehr ungewöhnliches Auftreten, mit Jeans und Hemd hinten aus der Hose. […] Die Herren waren alle sonst im Anzug mit Schlips […], das war schon mal sehr ungewöhnlich. Und dann hat er […] Dinge aus dieser Zeit [des Nationalsozialismus, MK] über die Art der Forschung und […] der Arbeiten [erzählt]. […] Das hat zu einem Eklat geführt, da haben Leute dann den Saal verlassen.*“

[27]

Gelegentlich wurde auch die aktive Auseinandersetzung mit der Geschichte gesucht. Einen Kulminationspunkt bildete eine Debatte um Hans Nachtsheim und den mit seinem Namen verbundenen Preis. In einem Gespräch wird von der Verleihung des nach Nachtsheim benannten Preises der Gesellschaft für Anthropologie und Humangenetik berichtet:

„*Der Preis trug nämlich den Namen Hans-Nachtsheim-Preis. Hans Nachtsheim war ja ein glühender Eugeniker und auch in gewisser Weise unverbesserlich. Er hat ja dann auch im Bundestag, also lange nach dem Krieg, das [GVeN] verteidigt. Das sei ja gar kein Nazi-Gesetz, im Gegenteil, die ganze Welt hätte Deutschland um dieses Gesetz beneidet. Er hat […] auch die Zwangssterilisierung noch verteidigt. […]*

*Ich wollte den Preis nicht annehmen. Weil er diesen Namen trug. […] Das heißt also ich stand vor diesem Dilemma entweder diesen Preis nicht anzunehmen oder eben anzunehmen und […] etwas zu sagen. Dann habe ich mich für das letztere entschieden weil das eben eine Chance war […]. Aufgrund dieser meiner Rede […] hieß der Preis von nächstem Jahr an dann nicht mehr Hans-Nachtsheim-Preis […]. Das hat offensichtlich der Fachgesellschaft die Augen geöffnet.*“

[28]

Nachtsheim war von 1941 bis 1945 als Leiter der Abteilung für experimentelle Erbpathologie am Kaiser-Wilhelm-Institut für Anthropologie, menschliche Erblehre und Eugenik tätig gewesen. Nach Alexander von Schwerins Analyse war Nachtsheim „*der mythisch-biologische Volksstaat kein Anliegen*“ und er lehnte Rassismus ab, andererseits profitierte er wissentlich von der Priorität, die biologische Forschung genoss. [29] Nachtsheim war nicht Mitglied der NSDAP. [29] Er arbeitete schwerpunktmäßig mit Kaninchen, führte aber 1943 auch Unterdruck-Experimente mit epilepsiekranken Kindern aus der Heil- und Pflegeanstalt Brandenburg-Görden durch. [30] Nach dem Ende des Zweiten Weltkriegs konnte Nachtsheim seine Karriere ab 1946 als Professor für Genetik an der Humboldt-Universität, später an der Freien Universität Berlin und Direktor des Max-Planck-Instituts für vergleichende Erbbiologie und Erbpathologie in Berlin-Dahlem fortsetzen. Er nahm u. a. an der Ausarbeitung der progressiven UN-Deklaration zum ‚Rasseproblem‘ teil. [29] Für das Verhalten deutscher Biowissenschaftler vor 1945 fand er überraschend deutliche Worte. So schrieb er 1947: „*Die deutsche Fachwissenschaft trifft – mit wenigen Ausnahmen – eine ähnlich schwere Schuld wie den deutschen Generalstab, der sich – unfaßlich für viele Deutsche – willig zum Handlanger der Verbrechen Hitlers machen ließ*.“ [29] Gleichzeitig setzte sich Nachtsheim auch in der Bundesrepublik weiter aktiv für Eugenik ein. Er wollte das GVeN nicht als nationalsozialistisches Unrechtsgesetz verstehen und forderte ein neues Gesetz zur Sterilisation aus eugenischer Indikation in Westdeutschland. [31] Noch 1963 sprach er von einer „*Pflicht zur Praktischen Eugenik*“. [32]

Diese ambivalente Haltung gestehen ihm so auch Zeitzeugen in ihrer Erinnerung zu, wenn es hier heißt, Nachtsheim habe eine „*Tendenz zu eugenischem Gedankengut*“ gehabt, „*was ich ihm aber nachgesehen habe*, [*weil*] *er so prägend gewesen ist* [*und*] *er eine herausragende Persönlichkeit in der wissenschaftlichen Geschichte gewesen ist*.“ [33]

Historische Reflexion wurde nach den Berichten aber auch jenseits des Nachtsheim-Preises von Nachfragen aus der breiteren Gesellschaft angestoßen. Ein Zeitzeuge berichtet:

„*Im Anschluss an meine Vorträge habe ich stets zu Diskussionen aufgerufen. Die Frage nach dem Unterschied zwischen der staatlich angeordneten Eugenik und der individuellen Entscheidung einer beratenen Frau nach pränataler Diagnostik musste häufig nochmals beantwortet werden. Ich hatte meinen eigenen Standort gefunden und konnte ihn in der Diskussion darlegen.*“

[34]

Ferner führte solches Erinnern zu einer Wahrnehmung von historisch bedingter Verantwortung, wie die folgende Aussage nahelegt:

„*Ich hatte einmal eine Familie zur Beratung, die sagte: ‚Wir möchten bitte, wenn alles abgeschlossen ist, dass alle, absolut alle Unterlagen vernichtet werden. Denn wir hatten zwei Familienangehörige, die in der Nazizeit umgebracht wurden.‘ Und natürlich akzeptiert man das sofort. Und das tut einem auch leid. Denn es war bis heute eine Belastung.*“

[35]

Neben der partikularen historischen Verantwortung als Humangenetiker*innen gaben einige Interviewte auch von sich aus einem Gefühl einer mehr universalen historischen Verantwortung Ausdruck. Diejenigen, die sich in dieser Weise geäußert haben, sind alt genug, um noch einen Teil des ‚Dritten Reichs‘ bewusst erlebt zu haben. Beispielsweise bemerkt ein Gesprächspartner: *„In der Tat habe ich als Medizinstudent versucht, Stalinismus und Nationalsozialismus zu verstehen. Habe auch entsprechende Bücher gelesen, bin bei beiden aber irgendwie stecken geblieben. Beides konnte ich nicht wirklich erklären, wie intelligente Länder zu so etwas kommen konnten*.“ [26]

## Diskussion und Ausblick

Alida Assmann hat mit Blick auf die westdeutschen Erinnerungskultur konstatiert, dass sich Formen kollektiver Erinnerung herausgebildet haben, die „*dunkle Kapitel*“ der Geschichte nicht mehr marginalisieren, *„sondern für diese Verantwortung übernehmen, indem sie sie im kollektiven Gedächtnis stabilisieren und ins kollektive Selbstbild integrieren*.“ Assmann spricht von einer *„sekundären Zeugenschaft der Gesellschaft in Form einer Erinnerungskultur, die von der Empathie und Solidarität mit den Opfern getragen ist und historische Verantwortung übernimmt*.“ [8] Eine solche Erinnerungskultur lässt sich auch in der deutschen Humangenetik finden. Stellungsnahmen der GfH, Veröffentlichungen von Akteur*innen des Fachs [36], [37], [38], [39], [40], [41], Geschichtspolitisches Handeln wie die Umbenennung des Hans-Nachtsheim-Preises und die Aussagen von Zeitzeug*innen machen deutlich, dass aus dem Geschichtsbewusstsein der deutschen Humangenetik eine historische Verantwortung erwachsen ist.

Dieser Prozess kam substantiell im Verlauf der 1980er Jahre in Gang – einer Dekade, in der die im Rahmen dieser Studie befragten Zeitzeug*innen aktiv waren und von der sie berichtet haben. Persönlich durch politisches Engagement im Nationalsozialismus oder für Eugenik in der Nachkriegszeit belastete Personen waren zu diesem Zeitpunkt im Fach kaum mehr wirkmächtig. Eine Kombination aus gesellschaftlichem und (medizin-)historischem Interesse von außen und der Schaffung einer professionellen Identität der deutschen Humangenetik, die verantwortungsvoll mit ihrem Erbe umgehen wollte, prägte ab diesem Zeitpunkt das Geschichtsbewusstsein. Historisch-kritische Veröffentlichungen lösten bei Humangenetiker*innen und in der Öffentlichkeit Diskussionen über Vergangenheit und Gegenwart des Fachs aus. Diese Erinnerungskultur führte wiederum zu Stellungnahmen der GfH und weiteren Publikationen, so dass hier von einer gegenseitigen Verstärkung von kulturellem und sozialem Gedächtnis gesprochen werden kann. Weitere Forschung hat das Potential zu zeigen, ob auch das Fach betreffende ethische Fragen und die Gründung der Deutschen Gesellschaft für Humangenetik 1987 von Geschichtsbewusstsein und historischer Verantwortung beeinflusst wurden.
